# Greater Number of Microglia in Telencephalic Proliferative Zones of Human and Nonhuman Primate Compared with Other Vertebrate Species

**DOI:** 10.1093/texcom/tgab053

**Published:** 2021-09-06

**Authors:** Elisa Penna, Christopher L Cunningham, Stephanie Saylor, Anna Kreutz, Alice F Tarantal, Verónica Martínez-Cerdeño, Stephen C Noctor

**Affiliations:** MIND Institute, School of Medicine, UC Davis, Sacramento, CA 95817, USA; Department of Psychiatry and Behavioral Sciences, School of Medicine, UC Davis, Sacramento, CA 95817, USA; Neuroscience Graduate Program, UC Davis, Davis, CA 95616, USA; Current Affiliation: Pittsburgh Hearing Research Center, Department of Otolaryngology, University of Pittsburgh, Pittsburgh, PA 15213, USA; Department of Psychiatry and Behavioral Sciences, School of Medicine, UC Davis, Sacramento, CA 95817, USA; Neuroscience Graduate Program, UC Davis, Davis, CA 95616, USA; Department of Pediatrics, School of Medicine, University of California at Davis, Davis, CA 95616, USA; Department of Cell Biology and Human Anatomy, School of Medicine, University of California at Davis, Davis, CA 95616, USA; California National Primate Research Center, University of California at Davis, Davis, CA 95616, USA; MIND Institute, School of Medicine, UC Davis, Sacramento, CA 95817, USA; Department of Pathology and Laboratory Medicine, Institute for Pediatric Regenerative Medicine, School of Medicine, UC Davis, Sacramento, CA 95817, USA; Shriners Hospital, Sacramento, CA 95817, USA; MIND Institute, School of Medicine, UC Davis, Sacramento, CA 95817, USA; Department of Psychiatry and Behavioral Sciences, School of Medicine, UC Davis, Sacramento, CA 95817, USA; Neuroscience Graduate Program, UC Davis, Davis, CA 95616, USA

**Keywords:** cortical development, microglial cells, neural precursor cells, proliferative zones

## Abstract

Microglial cells, the innate immune cells of the brain, are derived from yolk sac precursor cells, begin to colonize the telencephalon at the onset of cortical neurogenesis, and occupy specific layers including the telencephalic proliferative zones. Microglia are an intrinsic component of cortical germinal zones, establish extensive contacts with neural precursor cells (NPCs) and developing cortical vessels, and regulate the size of the NPC pool through mechanisms that include phagocytosis. Microglia exhibit notable differences in number and distribution in the prenatal neocortex between rat and old world nonhuman primate telencephalon, suggesting that microglia possess distinct properties across vertebrate species. To begin addressing this subject, we quantified the number of microglia and NPCs in proliferative zones of the fetal human, rhesus monkey, ferret, and rat, and the prehatch chick and turtle telencephalon. We show that the ratio of NPCs to microglia varies significantly across species. Few microglia populate the prehatch chick telencephalon, but the number of microglia approaches that of NPCs in fetal human and nonhuman primate telencephalon. These data demonstrate that microglia are in a position to perform important functions in a number of vertebrate species but more heavily colonize proliferative zones of fetal human and rhesus monkey telencephalon.

## Introduction

Key aspects concerning the generation and migration of cortical neurons in mammalian cerebral cortex have been well described. Birth dating experiments show that neural precursor cells (NPCs) in the mammalian cerebral cortex generate neurons and astroglia over a period ranging from days in the mouse (Caviness et al. 2003) and rat (Bayer and Altman 1991), to weeks in carnivores such as the ferret (Jackson et al. 1989; Noctor et al. 1997), and months in rhesus monkey (Rakic 1974) and human (Bayer et al. 1993; Bayer and Altman 2007; Malik et al. 2013).

Less is known concerning when microglial cells are generated since these cells appear to retain the capacity for division, hindering birth date and migration studies of microglia based on traditional thymidine analog labeling. Nonetheless, del Rio-Hortega proposed that microglia are derived from extra-cortical sites and enter the cerebral cortex during prenatal development (del Rio-Hortega 1919; del Rio-Hortega 1932). Research in the 1990s provided evidence that microglia are indeed extra-cortical and are derived from the yolk sac in chick, mouse, and rat (Ashwell 1991; Cuadros et al. 1993; Alliot et al. 1999), and this concept was confirmed through lineage and fate-mapping studies in mouse (Ginhoux et al. 2010; Schulz et al. 2012). Microglia begin populating the cerebral cortex at approximately the same time as the onset of cortical neurogenesis in mouse, rat, rhesus monkey, and human (Andjelkovic et al. 1998; Rezaie and Male 1999; Monier et al. 2006; Verney et al. 2010; Cunningham et al. 2013; Swinnen et al. 2013). Upon entering the prenatal cerebral cortex microglia populate specific layers including the germinal zones (Rezaie and Male 1999; Cunningham et al. 2013; Arno et al. 2014). After cortical cell genesis is complete microglia distribute evenly across the cortical wall.

Recent data show that in the prenatal brain microglia are an intrinsic component of cortical proliferative zones and are tightly integrated with NPCs (Barger et al. 2019). Each microglial cell near the lateral ventricle simultaneously contacts multiple mitotic NPCs in a cell-cycle-dependent manner (Noctor et al. 2019). In addition, both microglia and NPCs are tightly integrated with the vessels and filopodia of developing cortical vasculature in the rat ventricular zone (VZ; Penna et al. 2021). In our studies, we noted significant differences in the distribution of microglia between rat and rhesus monkey (Cunningham et al. 2013), and that rhesus monkey cortical proliferative zones are populated by 10-fold more microglia compared with rat at the same stages of development (Barger et al. 2019).

Given the large difference in the size of microglial cell populations we observed between rat and monkey, we analyzed the relative number of NPCs and microglial cells in the dorsal telencephalon of several vertebrate species. We quantified the number of Pax6^+^ and Tbr2^+^ NPCs, and microglial cells in the proliferative zones of prenatal human, rhesus monkey, ferret, rat, birds, and turtle telencephalon. We also compared the morphological phenotype of microglia in the proliferative zones and characterized basic interactions between microglia and NPCs across vertebrate species. We found similar morphological phenotypes of microglial cells across the vertebrate species that we examined. Microglial cells in telencephalic proliferative zones across species demonstrated a stereotypical “activated” morphological profile, and in each species, we observed microglia enveloping NPCs, as we have described in rat and rhesus monkey (Cunningham et al. 2013; Barger et al. 2019). We noted that there were more microglia relative to NPCs in the proliferative zones of the primate brain compared with the other vertebrate species in our sample. Taken together, these findings establish basic fundamentals concerning microglial colonization of developing vertebrate telencephalon, shed light on common functions of microglia across vertebrate species, and indicate that microglial colonization of the telencephalon occurred early in vertebrate evolution.

## Materials and Methods

### Animals

All animal procedures were approved by the UC Davis Institutional Animal Care and Use Committee of the University of California, Davis. The study utilized 8 timed pregnant adult rats, 15 embryonic rats, and 9 postnatal rats (Sprague Dawley). Embryonic day (E)16, E17, E18, E19, and E20 rats (*n* = 3 for each age stage), and postnatal day (P)0, P3, and P10 rats (*n* = 3 for each age stage) were transcardially perfused with 0.1 M phosphate-buffered saline pH 7.4 (PBS) followed by 4% paraformaldehyde in PBS (PFA). The brains were immediately removed after perfusion and postfixed by immersion into 4% PFA at 4 °C for 2 h, cryoprotected overnight at 4 °C in 30% sucrose buffer solution in preparation for tissue processing as previously described (Noctor et al. 2019). Chicken eggs (*Gallus gallus domesticus*, *n* = 24) were obtained from the UC Davis Animal Science Department Avian Facilities, Davis, CA. Eggs were incubated in a Hova-Bator thermal incubator. Embryos were removed from eggs; incubation day (designated “E”) chicken brains at E3, E6, E7, E8, E9, E10, E12 and E15 (*n* = 3 for each age stage) were fixed by direct immersion in 4% PFA for 24 h at 4 °C. Ferret tissue (*Mustela putorius furo*) was collected by the laboratories of Dr Francisco Clasca, Dr Sharon Juliano, and Dr Barbara Chapman, and sections of brain tissue were provided for this study as a generous gift. Studies also included fetal rhesus monkeys (*Macaca mulatta*, 50-, 65-, 80-, 100- and 150-day gestational age; term 165 ± 10 days). Female rhesus monkeys (*n* = 5) were time-mated and identified as pregnant by ultrasound according to established methods (Tarantal 2005). All animal procedures conformed to the requirements of the Animal Welfare Act. In addition, some sections of rhesus monkey brain tissue were a generous gift from Dr David Amaral (UC Davis MIND Institute). Fixed fetal human brain tissue was provided to this project as a generous gift from Dr Tomasz Nowakowski (UCSF). Tissue was sectioned in the coronal plane at 20 μm on a sliding microtome or cryostat.

### Immunohistochemistry

Immunostaining was performed as previously described (Penna et al. 2021). For monkey and human samples, antigen retrieval was performed on slide-mounted tissue by heating sections in 10 mM citrate buffer (pH 6.0) containing 10 mM citric acid (Fisher) and (v/v) 0.5% Tween-20 (Acros) to 95 **°**C for 15 min. All the species sections were blocked in buffer containing (v/v) 10% fetal donkey serum, 0.1% Triton X-100 (Acros) for a minimum of 1 h at room temperature (RT). Sections were incubated in primary antibody buffer containing primary antibodies, (v/v) 2% fetal donkey serum, 0.02% Triton X-100, overnight at RT. Sections were rinsed in 0.1 M PBS, then incubated for 1 h at RT in secondary antibody buffer, which contained secondary antibodies (v/v) 2% fetal donkey serum, 0.02% Triton X-100, (v/v), and DAPI 1: 1000 (Roche). Primary antibodies: mouse monoclonal anti-PAX6 (1:50, Abcam Cat#ab78545, RRID AB_1566562); rabbit polyclonal anti-PAX6 (1:100, BioLegend Cat#901301, RRID AB_2565003); rabbit polyclonal anti-TBR2/Eomes (1:100, Abcam Cat#ab23345, RRID AB_778267); rabbit polyclonal anti-Tbr2 (1:100, Millipore Cat#AB15894, RRID_10615604); goat polyclonal anti-Iba1 (1:250, Abcam Cat#ab5076, RRID AB_2224402); and *Lycopersicon Esculentum* (Tomato) Lectin (LEL) DyLight488 conjugated (5 μg/mL, Vector Laboratories Cat#DL-1174, RRID AB_2336404) and *L. Esculentum* (Tomato) Lectin (LEL) FITC conjugated (Invitrogen Cat# L32478) overnight at RT. Donkey anti-mouse/anti-rabbit/anti-goat secondary antibodies conjugated respectively to Alexa Fluor 647 (1:500 Invitrogen Cat#A-31571, RRID AB_162542), Alexa Fluor 594 (1:500 Invitrogen Cat#A-21207, RRID AB_141637), and Alexa Fluor 488 (1:500 Invitrogen Cat#A-11055, RRID AB_2534102). Sections were rinsed three times for 5 min in PBS and cover-slipped with Mowiol 4–88 mounting medium (Sigma-Aldrich) and glass coverslips (Fisher).

### Imaging

Sections were imaged on an Olympus FV1000 IX81 confocal microscope with a 40× water immersion objective (NA 0.8 Olympus). Section thickness was 20 µm for all the species, and image Z-stacks were acquired at 1.0 µm steps. Sections were imaged from the ventricular surface through the VZ and subventricular zone (SVZ) proliferative area to include all Tbr2^+^ cells for quantitative analysis, and from the ventricular surface to the pial surface for qualitative observations. Images were arranged in montages to demonstrate the distribution of labeled cells across the telencephalon (Adobe Photoshop).

### Quantitative Analysis of Total Pax6^+^, TBR2^+^, and Iba1^+^ Cells

Coronal sections of rat, ferret, rhesus monkey, and human cerebral cortex taken from the rostro-caudal level of the anterior commissure (AC), and coronal sections of chick and dove telencephalon taken at a similar rostro-caudal level, were quadruple stained with Pax6, Tbr2, Iba1 or LEL microglial cell markers, and DAPI and then imaged on a confocal microscope (Olympus FV1000 IX81). Z-stack series were obtained from tissue containing fully stained cells, included the same number of Z-steps (*n* = 6) and therefore the same volume of stained tissue. Figures show projected images prepared from the Z-stacks. Montages of cortical radial units were created in Photoshop (Adobe) by combining individual images from a series of overlapping high magnification (40×) Z-stack projection images. For each montage, a 200-μm-wide bin was created within the image that extended from the ventricular surface in the radial dimension through the extent of the proliferative zones—VZ, iSVZ, and oSVZ—to include all Tbr2^+^ cells (see Figs 8*B*,*C*). All Pax6^+^, Tbr2^+^, and Iba1^+^ cells were counted within the bin. All Pax6^+^, Tbr2^+^, Iba1^+^, and LEL^+^ cells were confirmed by DAPI staining and counted using the multipoint tool (Fiji-ImageJ, NIH) and tracking labeled cells through the Z-stacks. Only cells that possessed DAPI^+^ nuclei that were located within the quantification bin were counted for analysis. The number of positive cells per bin was compared across ages and species.

### IMARIS 3D Cell Surface Model

Microglia Iba1^+^ and Tbr2^+^ NPC cell 3D segmentation and 3D surface reconstruction and visualization in human samples were performed using IMARIS imaging software (IMARIS 9.6, Bitplane-Oxford Instruments).

## Results

**Figure 1:**
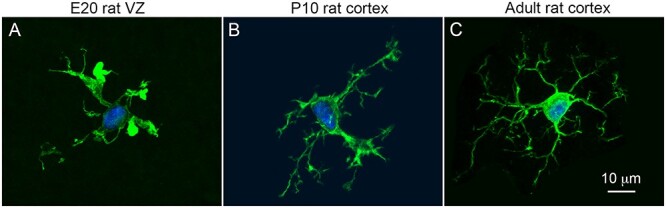
Microglial morphology in the telencephalon transitions from “activated” in the proliferative zones to the stereotypical resting surveillance phenotype in the healthy mature rat cerebral cortex. (*A*) Microglial cells (Iba1, green) in prenatal cortical proliferative zones typically have three or four short, thick processes displaying enlarged membranous extensions. (*B*) In the early, postnatal cortex microglial processes become thinner and extend more short branches. (*C*) Stereotypical phenotype of a microglial cell in the healthy adult rat cerebral cortex. These cells have five to six principal processes with finely branched processes. Blue, DAPI labeled nuclei. Scale bar in *C* refers to panels *A* and *B*.

Microglia are evenly distributed across the healthy adult cerebral cortex (Rezaie and Male 1999; Cunningham et al. 2013). Adult microglial somata are ~10 μm in diameter and typically extend five to six finely branched processes that radiate from the soma in all directions (Fig. 1*C*). Each cell and its processes occupy a sphere ~60 μm in diameter. The distribution and morphology of microglial cells in the prenatal brain are starkly different—these cells are concentrated in specific lamina including the meninges and proliferative zones and are morphologically distinct as well (Cunningham et al. 2013). Prenatal and early postnatal microglial somata are often larger, have an irregular shape, and have a smaller number of thick processes that extend from the soma (Figs 1*A*,*B*). Furthermore, microglia in cortical proliferative zones often exhibit complex morphologies and have processes that weave between and around neighboring cells in the densely packed proliferative zones as we have shown in previous publications (Barger et al. 2019; Noctor et al. 2019). Here, we compared the microglial cell population that colonized the proliferative zones of the telencephalon in several prehatch and prenatal vertebrate species with reference to the number of cortical NPCs to gain insights into evolutionary relationships between the two cell populations.

### Microglial Cell Morphology across Species

**Figure 2:**
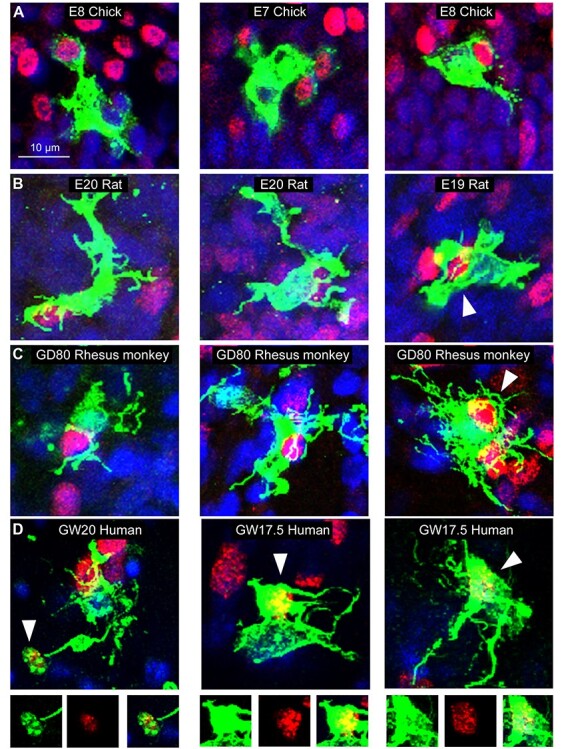
Microglia in the vertebrate cortical proliferative zones display an “activated” morphology and envelope NPCs. (*A*) LEL^+^ microglia (green) in the SVZ of the incubation day (E)7 and E8 chick telencephalon contact and envelope Tbr2^+^ NPC nuclei (red) in mesopallial and hyperpallial proliferative zones. (*B*) Examples show Iba1^+^ microglia (green) in the outer SVZ of embryonic day (E)19 and E20 rat cerebral cortex that contact and envelope Tbr2^+^ NPC nuclei (red). (*C*) Examples of Iba1^+^ microglia (green) in the outer SVZ of 80 days gestational age (GD80) rhesus monkey cerebral cortex contacting and enveloping Tbr2^+^ NPC nuclei (red). (*D*) Iba1^+^ microglia (green) in the outer SVZ of gestation week (GW)17.5 and GW20 fetal human cerebral cortex contact and envelope Tbr2^+^ NPC nuclei (red). Insets below each image present confocal microscope data showing that the Tbr2^+^ NPC nuclei were enveloped entirely within the Iba1^+^ cells and process of microglial cells. Blue, DAPI. Scale bar, 10 μm.

**Figure 3:**
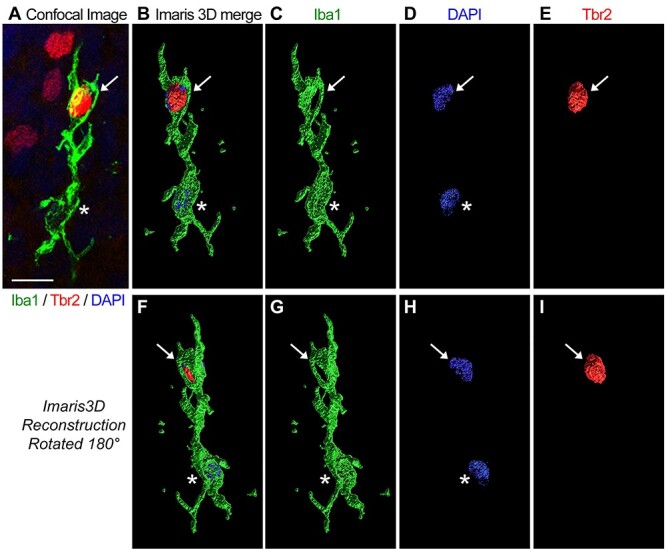
Microglia in fetal human cerebral cortex envelope NPCs. (*A*) A confocal image of an Iba1^+^ microglial cell (green) in the outer SVZ of gestation week (GW)20 fetal human cerebral cortex contacts and envelopes a Tbr2^+^ NPC nuclei (red, white arrow). The microglial cell nucleus (blue, DAPI) is indicated by the asterisk. (*B–E*) The same confocal data represented in 3D with Imaris software showing Iba1 (green) and Tbr2 (red) immunoreactivity and DAPI^+^ nuclei (blue). (*F–I*) The Imaris 3D representation rotated 180**°**. Scale bar, 10 μm. The Imaris 3D reconstruction of this fetal human microglial cell is also shown in Movie 1.

We analyzed the samples of telencephalon from chick, rat, ferret, rhesus monkey, and human fetuses. We obtained samples from developmental stages that encompassed peak periods of neurogenesis in the telencephalon for each species. Cell number was quantified in dorsal pallium and dorsal cerebral cortex (Fig. 8*B*,*C*). We first examined the morphological profile of microglial cells in the proliferative zones of the dorsal telencephalon in each vertebrate and observed similar morphologies across species. In all cases, microglia in proliferative zones displayed enlarged somata with thick processes that appeared to contact neighboring cells or were observed enveloping the Tbr2^+^ and/or Pax6^+^ nuclei of NPCs (Fig. 2). To better appreciate the close interactions between NPCs and microglia, and the envelopment of NPCs by microglia, we created a 3D representation of confocal Z-stack data obtained in fetal human cerebral cortex using Imaris software. As we have shown previously in rat and rhesus monkey (Cunningham et al. 2013; Barger et al. 2019), human microglial cells extend processes that closely envelope NPCs in cortical proliferative zones under normal conditions (Fig. 3 and Movie 1).

### Ratio of NPCs to Microglia

We next compared the number of microglia in telencephalic proliferative zones with reference to the number of NPCs. Analysis was performed in samples taken from developmental stages that encompassed neurogenesis and early gliogenesis. We constructed bins that were 200 μm wide, parallel to the ventricular surface, and extended perpendicular from the ventricular surface in the radial dimension through the entire extent of the proliferative zones. Bins encompassed the full complement of Tbr2^+^ cells in the radial dimension including the cohort of Tbr2^+^ cells located furthest from the ventricle (Fig. 8*B*,*C*). Thus, bins extended longer distances from the ventricle in samples taken from more advanced stages of cortical development in which proliferative zones were thicker (Martinez-Cerdeño et al. 2012).

**Figure 4:**
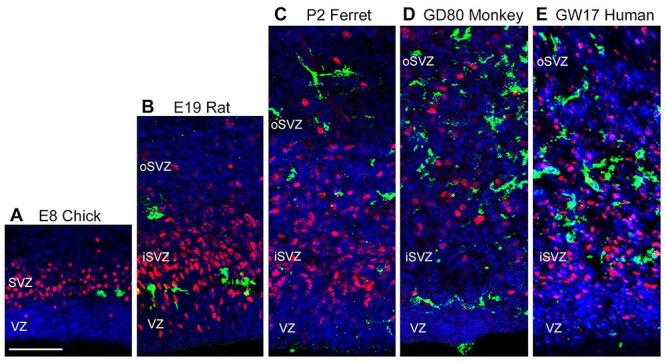
Relative distribution of microglial cells and Tbr2^+^ NPCs in proliferative zones of the prenatal telencephalon of chick, rat, ferret, monkey, and fetal human. (*A*) Incubation day (E)8 chicken mesopallium. A well-formed band of Tbr2^+^ cells (red) indicates the presence of the SVZ in chick mesopallium. LEL^+^ microglia (green) were sparse. (*B–E*) Coronal sections of the dorsolateral mammalian cerebral cortex taken at the rostro-caudal level of the anterior commissure. (*B*) Embryonic day (E)19 rat. The dense band of Tbr2^+^ cells (red) distinguishes the location of the inner SVZ (iSVZ) from the outer SVZ (oSVZ). Iba1^+^ microglia (green) were relatively sparse. (*C*) Postnatal day (P)2 ferret. Dense and diffuse bands of Tbr2^+^ cells mark the iSVZ and oSVZ, respectively. Iba1^+^ microglia (green) have a comparable distribution to that in rat. (*D*) 80 days gestational age (GD80) rhesus monkey fetus. A higher density of Iba1^+^ microglia (green) was colocalized with Tbr2^+^ cells (red) in the iSVZ and oSVZ of fetal monkey brain compared with ferret and rat. (*E*) Gestation week (GW)17 fetal human. Density of Iba1^+^ microglia (green) in the iSVZ and oSVZ in fetal human was similar to that observed in the fetal rhesus monkey. Microglia were observed contacting and enveloping Tbr2^+^ cells (red). Scale bar in *A* = 50 μm and applies to all panels.

### Birds and Turtle

#### Chick

**Figure 5:**
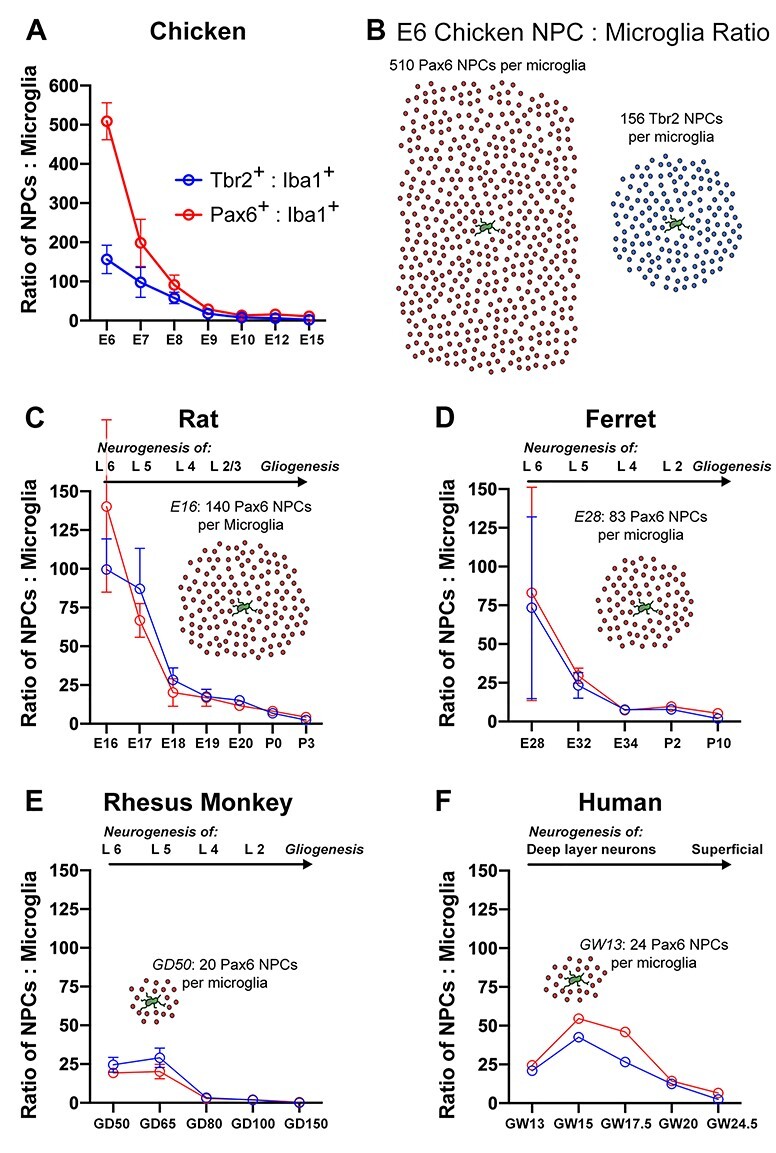
Graphical and schematic comparison of the ratio of NPCs to microglia in proliferative zones of dorsal telencephalon across prenatal vertebrate species. Cortical neurogenesis in fetal human and nonhuman primate begins with far more microglia in the telencephalic proliferative zones compared with other vertebrates. (*A*) The ratio of Pax6^+^ NPCs (red line) and Tbr2^+^ NPCs (blue line) to microglia in the proliferative zone of chick mesopallium is plotted at incubation day (E) 6, E7, E8, E9, E10, E12, and E15. The ratio was much higher in chicken than at any stage of mammalian neurogenesis included in this study—over 500 Pax6^+^ NPCs per single microglial cell at E6. The ratio quickly dropped and by E15 was 10:1 Pax6^+^ NPCs per microglia and 2:1 Tbr2^+^ NPCs per microglia. (*B*) Schematic representation of the number of NPCs for each microglial cell in the proliferative zone of the prenatal chick mesopallium. In the E6 chick, there were 510 Pax6^+^ NPCs (red) and 156 Tbr2^+^ NPCs (blue) for each microglial cell (green). (*C*) The ratio of Pax6^+^ NPCs (red line) and Tbr2^+^ NPCs (blue line) to microglia in the proliferative zones of rat dorsolateral cerebral cortex is plotted at embryonic day (E)16, E17, E18, E19, E20, postnatal day (P)0, and P3. Timeline at the top of the graphs indicates which cortical layer neurons were being generated at each stage of development. In some species, gliogenic stages of development were included in the analysis. The ratio of NPCs to microglia at E16 (layer 6 neurogenesis) was 140 Pax6^+^ NPCs per microglia and 100 Tbr2^+^ NPCs per microglia but decreased rapidly so that by E20 (layer 2/3 neurogenesis) the ratio was 11 Pax6^+^ NPCs per microglia and 15 Tbr2^+^ NPCs per microglia. The ratio reached parity (1:1) between P3 and P10 after completion of layer 2 neurogenesis. The inset schematic depicts the ratio of Pax6^+^ NPCs per microglial cell in rat cortical proliferative zones at E16. (*D*) The ratio of NPCs to microglia in ferret cortical proliferative zones is plotted at E28, E31, E34, P2, and P10. The ratio at E28 (layer 6 neurogenesis) started lower than in rat (83 Pax6^+^ NPCs and 73 Tbr2^+^ NPCs per microglial cell) and reached parity (1:1) after completion of layer 2 neurogenesis between P2 and P10. The inset schematic depicts the ratio of Pax6^+^ NPCs per microglial cell in ferret cortical proliferative zones at E28. (*E*) The ratio of NPCs to microglia in fetal rhesus monkey cortical proliferative zones is plotted at gestational day (GD)50, 65, 80, 100, and 150. The ratio at GD50 (layer 6 neurogenesis) started lower than in ferret (20 Pax6^+^ NPCs and 24 Tbr2^+^ NPCs per microglial cell) and reached parity after completion of layer 2 neurogenesis between GD100 and GD150. The inset schematic depicts the ratio of Pax6^+^ NPCs per microglial cell in rhesus monkey cortical proliferative zones at GD50. (*F*) The ratio of NPCs to microglia in fetal human cortical proliferative zones is plotted from gestation week (GW)13 to GW24.5, stages that include neurogenesis for both deep and superficial cortical layers (Malik et al. 2013; Arshad et al. 2016). The ratio of NPCs to microglia at GW13 was similar to that in monkey at GD50 during layer 6 neurogenesis (24 Pax6^+^ NPCs and 20 Tbr2^+^ NPCs per microglial cell) and decreased to 2:1 by GW24.5. The inset schematic depicts the ratio of Pax6^+^ NPCs per microglial cell in human cortical proliferative zones at GW13.

We quantified microglial and NPC cell number in coronal sections of chick telencephalon from eggs at incubation day (E)3, E6, E7, E8, E9, E10, E12, and E15. We examined mesopallial (MP) and hyperpallial (HP) proliferative zones. At E3, Pax6^+^ cells were present in both structures, and only a few Tbr2^+^ cells were observed in the MP. We did not observe microglial cells in either structure at this stage. At E6, microglial cells appeared in proliferative zones. The ratio of NPCs to microglia was 510:1 for Pax6^+^ cells and 156:1 for Tbr2^+^ cells. The number of Tbr2^+^ cells increased steadily across stages in both the MP and HP telencephalon, and there were more Tbr2^+^ cells than Pax6^+^ cells in MP proliferative zones at E7, E8, and E9. From E9 through E15, the number of microglia steadily increased and the number of NPCs in the counting bins declined. Consequently, by E15, the ratio of NPCs to microglia had fallen to 10:1 for Pax6^+^ cells and 2:1 for Tbr2^+^ cells (Fig. 5*E*).

#### Dove and Turtle

We analyzed coronal sections of telencephalon from a single stage each of mourning dove (incubation day E5 as per Muller et al. 1984), and turtle (stage 20 as per Clinton et al. 2014). Pax6^+^ and Tbr2^+^ cells were present in the proliferative zones of both species, but there were noticeably fewer Tbr2^+^ cells than were observed in the chick telencephalon. There were also fewer microglial cells in the proliferative zones of these species relative to chick. Consequently, the ratio of NPCs to microglia was substantially higher for Pax6^+^ cells in these species compared with other vertebrates included in this study—1096:1 for dove and 853:1 in E20 turtle. A thin but well-defined band of Tbr2^+^ cells was present superficial to the VZ in both species, as we have previously noted for turtles (Martinez-Cerdeno et al. 2016). The ratio of Tbr2^+^ NPCs to microglia was lower in both species, 68:1 in the dove and 106:1 in the turtle sample.

### Mammals

In mammalian species, we analyzed cell number in the dorsal telencephalon, in coronal sections at the rostro-caudal level of the AC. Data are displayed relative to the specific layer of cortical neurons being generated at that point as per published birth-dating studies (e.g., in ferret: Noctor et al. 1997).

#### Rat

We analyzed cell number in prenatal rat cerebral cortex on embryonic days (E)16 through E20 and on postnatal days (P)0, P3, and P10. We have previously shown that microglia begin colonizing rat cortical proliferative zones at the onset of neurogenesis and more densely colonize proliferative zones at the end of neurogenesis (E20) (Cunningham et al. 2013). At E16 during layer 6 neurogenesis (Bayer and Altman 1991), there were more Pax6^+^ than Tbr2^+^ cells. Quantification showed 140:1 Pax6^+^ cells per microglia and 100:1 Tbr2^+^ cells per microglia. The number of Tbr2^+^ cells increased between E17 and E20 and there were more Tbr2^+^ cells than Pax6^+^ cells at those stages. There was also a marked increase in the number of microglial cells that had colonized proliferative zones. Consequently, at E20, the ratio of NPCs per microglial cell decreased to 11:1 for Pax6^+^ cells and 15:1 for Tbr2^+^ cells per microglia. The NPC to microglial cell ratio was ~ 7:1 on the day of birth (P0); ~ 3:1 at P3; the ratio reached parity between P3 and P10, and by P10 the ratio was less than 1, indicating that there were more microglial cells than NPCs by that stage of development (Fig. 5*A*).

#### Ferret

We quantified cell number in perinatal ferret cerebral cortex on E28, E31, E34, P2, P10, and P18. Stages E28–P2 represent neurogenesis of deep through superficial layers in somatosensory cortex, and P10–P18 represent the postneurogenic stages of gliogenesis (Noctor et al. 1997). Throughout the stages of cortical development that we examined, there were similar numbers of Pax6^+^ and Tbr2^+^ cells per radial bin. At E28, which represents neurogenesis of layer 6 neurons in ferret somatosensory cortex (Noctor et al. 1997), there were very few microglia (1–3), and the ratio of NPCs to microglia was 83:1 for Pax6^+^ cells and 73:1 for Tbr2^+^ cells per microglial cell. As in rat, the number of microglial cells increased as ferret cortical development proceeded. Consequently, the ratio of NPCs per microglia steadily decreased to 10:1 for Pax6^+^ cells and 8:1 for Tbr2^+^ cells per microglia at P2, which represents the end of layer 2 neurogenesis in somatosensory cortex (Noctor et al. 1997). At P10, the ratio fell further to 5:1 for Pax6^+^ cells and 2:1 for Tbr2^+^ cells. The ratio reached parity between P10 and P18 (Fig. 5*B*).

#### Rhesus Monkey

We next quantified NPC and microglial cell populations in fetal rhesus monkey at 50, 65, 80, 100, and 150 days gestational age. The period of development from 50 days gestation (late first trimester) through 100 days (late second trimester) represents the majority of cortical neurogenesis in the occipital lobe (Rakic 1974), and 150 days gestation (near term) represents postneurogenic gliogenesis. We have previously shown that microglia begin to colonize cortical proliferative zones in fetal rhesus monkey at the onset of neurogenesis (50 days gestation) and densely colonize proliferative zones at the end of neurogenesis (100 days gestation) (Cunningham et al. 2013). At each stage of development that we examined, there was a similar number of Pax6^+^ and Tbr2^+^ cells in the radial bins. At 50 days gestational age, which represents neurogenesis of layer 6 neurons in rhesus monkey visual cortex (Rakic 1974), there were significantly more microglia in the proliferative zones compared with embryonic rat at the same stage of neurogenesis, as we have previously reported (Barger et al. 2019). Accordingly, the ratio of NPCs to microglia at the onset of cortical neurogenesis was much lower in rhesus monkey than we observed in rat or ferret: 20:1 for Pax6^+^ cells and 25:1 for Tbr2^+^ cells per microglial cell. While the number of NPCs increased in the rhesus monkey SVZ (Martinez-Cerdeño et al. 2012), the number of microglial cells also increased as development proceeded. Consequently, the ratio of NPCs to microglia decreased to ~ 3:1 for both Pax6^+^ and Tbr2^+^ cells by 80 days gestational age, which represents layer 4 neurogenesis, and ~2:1 at 100 days, which represents the end of layer 2 cortical neuron genesis in rhesus monkey visual cortex (Rakic 1974). The ratio of NPCs to microglia reached parity between 100 and 150 days of gestation (Fig. 5*C*).

#### Human

**Figure 6:**
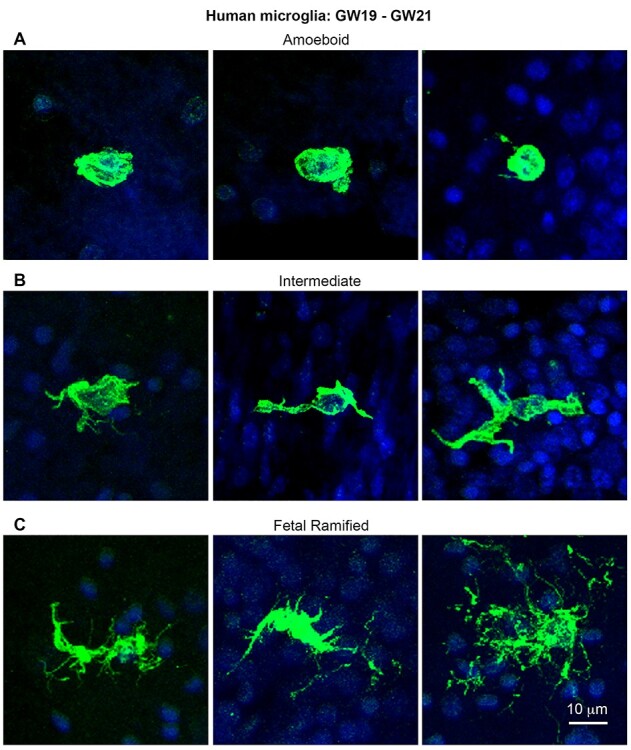
Morphological phenotype of microglia in proliferative zones of fetal human dorsal cerebral cortex. (*A*) Examples of microglial cells (Iba1, green) with “amoeboid” morphology present in cortical proliferative zones. Amoeboid cells were rare in fetal human cortex. (*B*) Microglia with an intermediate phenotype were common in the cortical proliferative zones. These cells usually had 1 to 3 µm thick processes extending from the soma and a few smaller branches. (*C*) Fetal ramified microglia were also common in cortical proliferative zones. These cells extended several thick processes from the soma and often possessed numerous fine processes. However, fetal ramified cells lacked the phenotypical symmetry of ramified cells observed in mature healthy cortex as shown in Figure 1*C*. Blue, DAPI. Scale bar in *C* refers to *A* and *B*.

We next examined NPC and microglial cell number in the proliferative zones of fetal human with tissue samples obtained from gestation week (GW) 13, 15, 17.5, 20, and 24.5 fetal neocortex. These stages do not encompass the entirety of cortical neurogenesis but represent the stages of both deep and superficial layer neurogenesis in neocortex (Malik et al. 2013; Arshad et al. 2016). Microglia begin colonizing the fetal human cortex by GW6/7 (Andjelkovic et al. 1998; Monier et al. 2006) and concentrate in specific lamina including the telencephalic proliferative zones (Rezaie and Male 1999; Cunningham et al. 2013). At each of the five fetal human stages included in this study, we observed microglia with a range of morphological phenotypes from amoeboid to more ramified (Fig. 6*A*–*C*). Amoeboid cells were rare in the fetal cortex—the vast majority of microglia exhibited fetal ramified or intermediate phenotypes. Fetal ramified cells had more processes than both the amoeboid and intermediate cell types but were distinct from ramified cells we observed in mature, healthy cerebral cortex (Fig. 1*C*).

**Figure 7:**
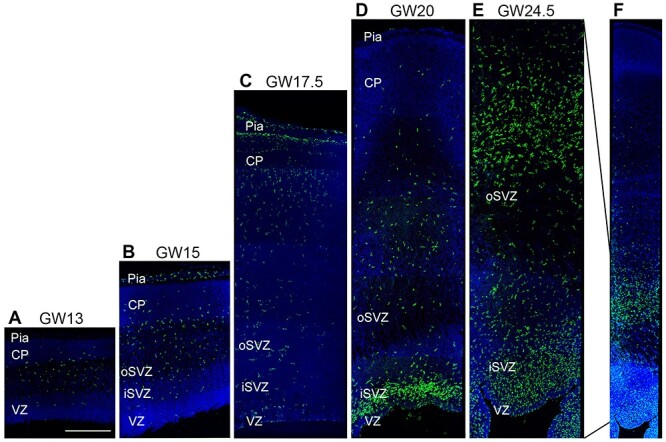
Distribution of microglial cells in fetal human dorsal cerebral cortex from gestation week (GW)13 through GW24.5. (*A–C*) Microglial cells (Iba1, green) were sparse in the samples of fetal human cortex between GW13 and GW17.5. Microglia were concentrated in the pial meninges of sample tissue sections that included meninges. (*D*) At GW20, microglial cells were concentrated in proliferative zones near the lateral ventricle in dorsal cortex as we have shown in fetal nonhuman primates (Cunningham et al. 2013). (*E*) At GW24.5, microglia densely populated the inner and outer SVZ (iSVZ, oSVZ), where NPCs undergo neurogenic divisions. Scale bar in *A* = 500 μm and applies to panels *A–E*, which are shown at the same scale. Panel *F* shows the entire cortical wall at GW24.5. Blue, DAPI.

In the GW13–17.5 human tissue samples, microglia were sparsely distributed across the wall of the telencephalon (Fig. 7*A*–*C*). At GW20, a dense band of microglia was observed near the VZ/SVZ interface, and in the GW24.5 sample, microglia were observed to densely populate the VZ, iSVZ, and oSVZ of fetal human cortex (Fig. 7*D*–*F*). This pattern closely resembled the pattern of microglial colonization of fetal rhesus monkey cortex in which microglia densely colonize fetal proliferative zones near the end of neurogenic stages of cortical development (Cunningham et al. 2013). The ratio of NPCs to microglia in the fetal human samples followed the same trend we observed in rhesus monkey—the ratio of NPCs to microglia was much lower than that observed in either rat or ferret. At GW13, the ratio of NPCs to microglia was 24:1 for Pax6^+^ cells and 21:1 for Tbr2^+^ cells per microglial cell. At GW20, the ratios decreased to 14:1 for Pax6^+^ cells and 12:1 for Tbr2^+^ cells per microglia, and at GW24.5, fell further to 7:1 for Pax6^+^ cells and 2:1 for Tbr2^+^ cells per microglia (Fig. 5*D*).

## Discussion

The timing of cortical neurogenesis has been accurately determined for multiple mammals including mouse, rat, hamster, rhesus monkey, ferret, and others (Angevine and Sidman 1961; Berry and Rogers 1965; Shimada and Langman 1970; Rakic 1974; Jackson et al. 1989; Bayer and Altman 1991; Noctor et al. 1997). The timeline of cortical neurogenesis in human has been estimated at approximately GW6–GW20 based on comparison with data from rat (Bayer et al. 1993), but more recent data suggest that the production of excitatory neurons continues into the third trimester and that production of cortical interneurons may persist beyond GW35 (Malik et al. 2013; Arshad et al. 2016). However, with few exceptions (e.g., Spalding et al. 2005), we know comparatively less about genesis of macro- and microglial cell populations in the prenatal cerebral cortex since many glia retain the capacity for division after birth (Reu et al. 2017). Given the comparative lack of data on microglial cell ontogeny, we quantified microglial cell number with respect to NPC number in the developing telencephalon to broaden our understanding of microglial cell biology.

### Microglia Colonize Telencephalic Proliferative Zones in Prenatal Vertebrates

We found that microglia colonized the proliferative zones of the prenatal telencephalon in each vertebrate we examined—one reptile, two species of bird, one rodent, one carnivore, one nonhuman primate, and fetal human—and in each case, we found similar outcomes at the onset of neurogenesis. The arrival of microglia in proliferative zones at a similar time in brain development in our sample of vertebrate species, including bird and reptile, indicates that this feature of microglial ontogeny occurred early in the evolution of telencephalic structures. Further supporting this concept, we observed microglia interacting with NPCs in bird and turtle in the same manner we observed in mammals (Fig. 2). The alternative case that this feature evolved independently in multiple vertebrate classes is possible, but less likely. Future studies that employ a broader sample of vertebrate species will provide deeper understanding of the functional and evolutionary relationships between NPCs and microglia in prenatal proliferative zones.

Microglial cells are similar in size in healthy cerebral cortex of many mammals. Likewise, we found that prenatal microglia displayed similar morphological properties in the prenatal proliferative zones of a variety of vertebrate species. The prenatal microglia all displayed short, relatively thick processes that are associated with exemplary “activated” microglia (Fig. 1*A*,*B*). In each species, we found examples of microglia with basic morphological phenotypes that we termed fetal ramified, intermediate, and amoeboid (Fig. 6). The amoeboid cells were rare in each species, and intermediate and fetal ramified cells made up the vast majority of microglia in cortical proliferative zones. Fetal ramified cells (Figs 2, 3, and 6*A*–*C*) differed substantially from ramified cells that are present in the healthy adult mammalian cortex (Fig. 1*C*). While the morphology of microglial cells appears to be correlated with laminar position in the fetal cerebral cortex and intercellular connectivity, the functional significance of microglial cell morphology in the fetal brain is not yet clear.

### Microglia Interact with Cortical Precursor Cells in Prenatal Vertebrates

In each species, even those with comparatively few microglia in the forebrain, we observed microglia contacting, extending phagocytic cups toward, and enveloping Tbr2^+^ and Pax6^+^ NPC nuclei in the proliferative zones (Figs 2 and 3). Our previous work has shown that this cellular behavior occurs in prenatal proliferative zones of the normally developing brain and is more common in fetal rhesus monkey and human (Cunningham et al. 2013), perhaps due to the higher density of microglia in these species (Barger et al. 2019). Phagocytosis is but one outcome upon contact between NPCs and microglia. We have shown that microglia are highly interconnected with mitotic NPCs, contacting both the pial fiber and soma of NPCs that divide at the surface of the lateral ventricle (Noctor et al. 2019). These interactions indicate the potential for microglia to impact the cellular behavior of NPCs.

### The Ratio of NPCs to Microglia Varies across Vertebrates

We observed clear differences in the number of microglia that populate proliferative zones across species at comparable stages of cortical neurogenesis (Fig. 5). Since there are also significant differences in the number of NPCs in proliferative zones between species, we quantified the ratio of NPCs to microglia across stages of cortical development. Data were registered with reference to the stages of cortical layer formation. For example, the ratio of NPCs to microglia in the 50 days gestation rhesus monkey (late first trimester) was compared with that in E16 rat and E28 ferret since cortical layer 6 neurons are generated at that stage in those species (Rakic 1974; Bayer and Altman 1991; Noctor et al. 1997).

Among mammalian species included in our study, the ratio of NPCs to microglia at the onset of layer 6 genesis was highest in rats (140 Pax6^+^ and 100 Tbr2^+^ per single microglia), followed by ferrets (83 Pax6^+^ and 73 Tbr2^+^ per microglia). In contrast, the ratio of NPCs to microglia was significantly lower in rhesus monkey during layer 6 neurogenesis (19 Pax6^+^ and 25 Tbr2^+^ per single microglia) and in the earliest sample of fetal human tissue (24 Pax6^+^ and 21 Tbr2^+^ per single microglia). From that point of prenatal development moving forward in each species, the number of microglia increased, and the number of NPCs decreased such that the ratio of NPCs to microglia approached parity near the end of cortical neurogenesis. In most cases, the ratio inverted to less than one after neurogenesis. The decreased ratio reflects the depletion of NPCs at the end of cortical neurogenesis and a continued increase in the cortical microglia population (Fig. 5).

Cortical proliferative zones are substantially thicker in human and rhesus monkey than in mouse, rat, or ferret (Smart et al. 2002; Fietz et al. 2010; Hansen et al. 2010; Martinez-Cerdeño et al. 2012). For example, the combined thickness of the VZ and SVZ in rhesus monkey is up to 10 times that in rat, and the proliferative zones harbor significantly more NPCs (Martinez-Cerdeño et al. 2012). In the present study, we recorded an increased number of microglia in the quantification bins of rhesus monkey and human. However, the number of microglia in primates did not increase proportionate to the increased thickness of cortical proliferative zones, but instead resulted from an increased density of microglia (Cunningham et al. 2013). Our previous work has shown that microglial cell density in rhesus monkey proliferative zones is at least 10 times greater compared with rat at the same stage of cortical neurogenesis (Barger et al. 2019). Here, we show comparable density of microglia in cortical proliferative zones of fetal human brain. Consequently, the lower ratio of NPCs to microglial cells in rhesus monkey and human cerebral cortex signified the increased number of microglia in primate proliferative zones (Fig. 4). We previously reported that the density and position of microglial cells within the fetal rhesus monkey SVZ varied markedly in neighboring cortical areas of the occipital lobe but did not differ significantly drastically across cortical areas in the rat (Cunningham et al. 2013). The significance of differences in microglial distribution across species, and across cortical areas within subjects, has not yet been determined.

### A Double-Edged Sword

The different ratio of NPCs to microglia we observed across species may reflect different timing in the onset of microglial colonization of the telencephalon with respect to cortical histogenesis, or distinct proliferative behaviors of microglial cells once they have arrived in the cerebral cortex. Different timing of microglial cell arrival in the developing brain could reasonably be expected to exert differential effects on brain development across species. Published literature reveals a consensus that microglia play important roles in the early postnatal brain and contribute to a wide array of developmental programs including cortical layer formation, axon pathfinding, synapse development, and synapse maintenance (Paolicelli et al. 2011; Schafer et al. 2012; Ueno et al. 2013; Squarzoni et al. 2014). Our work has demonstrated that from early stages of cortical neurogenesis, microglia are highly interactive and intrinsically connected with key cells and structures in the prenatal brain (Cunningham et al. 2013; Barger et al. 2019; Noctor et al. 2019; Penna et al. 2021). Earlier colonization of the primate telencephalon by microglia may allow these cells to influence a wider array of developmental programs during cortical formation and to make a more significant contribution in shaping development of the prenatal brain. However, this potential developmental benefit may come with risks. Fetal microglia rapidly respond to changes in their local environment, injury, and extrinsic factors introduced through maternal exposure to pathogens (Davalos et al. 2005; Antonson et al. 2019; Gurung et al. 2019). The capacity for rapid microglial response increases the potential for pathogens to impact the typical trajectory of brain development via involvement of microglial cells, especially given the interconnectivity of microglia with vital components in telencephalic proliferative zones. Supporting this concept, we have found that fetal exposure to the Zika virus in the rhesus monkey produces profound changes in those key components. At only 3 weeks after Zika inoculation microglial distribution was altered, with these cells collected in large heterotopic clusters throughout cortical proliferative zones. The microglial clusters were associated with disturbed distribution of Tbr2^+^ NPCs, enlarged blood vessels, and a thinner cortical plate, and these altered parameters persist at 3 months postexposure (Tarantal et al. 2021).

**Figure 8:**
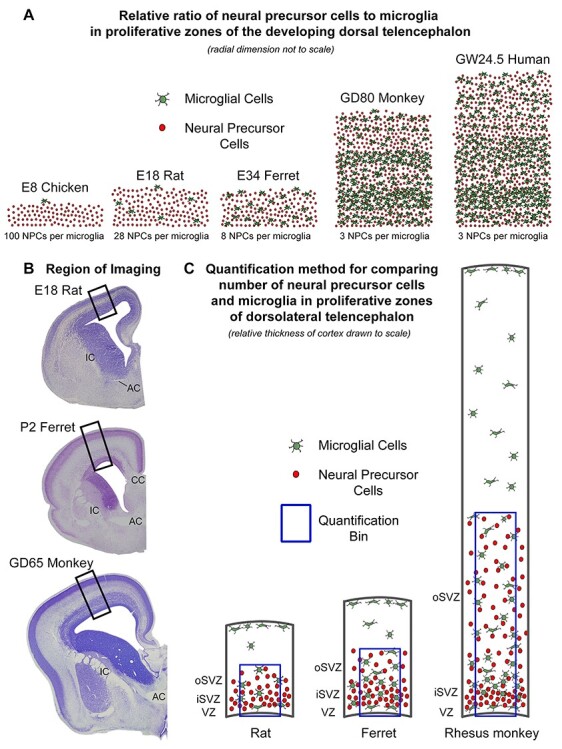
(*A*) Schematic representations depict the relative number of NPCs (red) and microglia (green) in proliferative zones of the dorsal telencephalon of prenatal vertebrates at comparative stages of neurogenesis—layer 4 cortical neurons are generated in the embryonic day (E)18 rat, E34 ferret and gestation day (GD)80 rhesus monkey dorsal cerebral cortex. Gestation week (GW)24.5 fetal human exhibits a similar density of microglia in proliferative zones compared with GD80 rhesus monkey. Schematic drawings demonstrate the notable difference in microglial cell number and density in proliferative zones of dorsal telencephalon of chick and rat compared with human and nonhuman primates. Thickness of proliferative zones not shown to scale. (*B*) Representative images of Nissl-stained tissue cut in the coronal plane at the rostro-caudal level of the anterior commissure (AC) from embryonic day (E)18 rat, postnatal day (P)2 ferret, and gestation day (GD)65 rhesus monkey. Images of immunostained tissue from each species were obtained from dorsal cerebral cortex in radial bins that extended from the ventricle to the pial meninges (black box). (*C*) Schematic showing method for quantifying the relative number of NPCs and microglial cells in cortical proliferative zones across species. Representative examples show developing rat, ferret, and rhesus monkey drawn to scale. Analysis was carried out in proliferative zones of dorsolateral cerebral cortex or pallium of each species. Quantification bins (blue) were 200 μm wide and extended from the ventricular surface through the proliferative zones to include all Tbr2-expressing cells.

### Summary

The data presented here show that microglia are present in the prenatal proliferative zones of all vertebrate species included in our study, suggesting that this feature arose early in vertebrate evolution. We show that the relative number of microglia and NPCs at specific stages of prenatal brain development varies across species, with more microglia per NPCs in the fetal nonhuman primate and fetal human telencephalon (Fig. 8). The comparatively early arrival of microglia in proliferative zones of fetal primate telencephalon, and the larger number of microglia present throughout development, particularly during cortical neuron production, provides opportunity for microglia to play a more prominent role in cellular genesis and maturation in primates, and supports the concept that microglial cells contribute to the formation of the telencephalon from early stages of development. The data also suggest that microglia may be one conduit between pathogen exposure and atypical outcomes in primate neurodevelopment. Future studies on intercellular communication between cells and structures in prenatal proliferative zones will further define the functional roles of microglial cells in the prenatal brain under normal conditions and with respect to neurodevelopmental disorders.

## Supplementary Material

Figure_3_Supplemental_Movie_tgab053
